# Pendelluft in patients with acute respiratory distress syndrome during trigger and reverse triggering breaths

**DOI:** 10.1038/s41598-023-49038-9

**Published:** 2023-12-13

**Authors:** Wei-Chieh Lin, Pei-Fang Su, Chang-Wen Chen

**Affiliations:** 1 Section of Critical Care Medicine, Department of Internal Medicine, National Cheng Kung University Hospital, College of Medicine, National Cheng-Kung University, Tainan, Taiwan; 2 Department of Statistics, National Cheng Kung University, Tainan, Taiwan

**Keywords:** Respiratory distress syndrome, Respiration

## Abstract

Pendelluft, the shift of air from non-dependent to dependent lung regions, is known to occur during active breathing in ventilated patients. However, information about pendelluft in ARDS patients under assisted mechanical ventilation is limited. In this prospectively collected and retrospectively analyzed study, we combined electrical impedance tomography and respiratory mechanics monitoring to quantitatively examine pendelluft in trigger and reverse triggering breaths in 20 mechanically ventilated patients with ARDS during the transition from controlled to active breaths under volume-cycled ventilation. Besides the 10 resting breaths in each patient, 20% of the counted active breaths were selected based on three levels of esophageal pressure swing (∆P_es_): low (< 5 cm H_2_O, breaths = 471), moderate (≥ 5, < 10 cm H_2_O, breaths = 906), and high effort (≥ 10 cm H_2_O, breaths = 565). The pendelluft response to breathing efforts was significantly greater in trigger breaths than in reverse triggering breaths (*p* < 0.0001). Based on the pendelluft-∆P_es_ slope (ml/cmH_2_O), there were two distinct patterns of effort-related pendelluft (high vs. low pendelluft group). For trigger breaths, the high pendelluft group (n = 9, slope 0.7–2.4 ml/cmH_2_O) was significantly associated with lower peak airway/plateau pressure and lower respiratory system/lung elastance than the low pendelluft group (n = 11, slope − 0.1 to 0.3 ml/cmH_2_O). However, there was no difference in respiratory mechanics between high and low pendelluft groups for reverse triggering breathes. The use of ∆P_es_ to predict pendelluft was found to have a low positive predictive value.

Acute respiratory distress syndrome (ARDS) is characterized by an increased lung inhomogeneity^1^. In ARDS patients under controlled ventilation, tidal ventilation distribution is usually diverted to the non-dependent lung region due to less aeration in the gravitationally dependent lung regions^2^. After switching to assisted mechanical ventilation, redistribution of ventilation to the dependent lung region occurs as the dependent part of the diaphragm moves the most^3^. Appropriate breathing effort may improve ventilation homogeneity. However, due to the heterogeneous distribution of lung inflammation and the different properties of atelectatic or fluid-filled lung tissue compared to well-aerated lung tissue in ARDS, negative pleural pressure generated by the diaphragm is not equal over the whole lung and is usually more negative over the dependent lung region where atelectasis tends to be greater^4^. In 2013, Yoshida et al. demonstrated the existence of a shift of air from non-dependent to dependent lung regions, known as pendelluft, during spontaneous breathing in pigs with acute lung injury^4^. Pendelluft is a phenomenon detected using electrical impedance tomography (EIT) that may cause local overstretching of the lungs^4^. This rapid inflation-deflation phenomenon is injurious, and it is desirable to reduce the intensity of spontaneous breathing effort once significant pendelluft is present^5^. Although the degree of pendelluft is supposed to be effort-related in trigger and reverse triggering breaths^4,6^, limited clinical observations have revealed that patterns of pendelluft may vary in mechanically ventilated patients who are ready for weaning^7^. Information about pendelluft in ARDS patients under assisted mechanical ventilation is lacking but should be of great clinical interest.

Using esophageal pressure and EIT monitoring, we prospectively collected data and conducted a retrospective analysis of an observational study in moderate to severe ARDS patients during the transition from controlled to spontaneous breathing following the termination of neuromuscular blocking agents. We documented respiratory mechanics and observed pendelluft variations at rest and during progressively increasing breathing effort as spontaneous breathing gradually resumed following the discontinuation of neuromuscular blocking agents. We theorized that pendelluft may occur in ARDS patients with increasing breathing effort, following the transient termination of neuromuscular blocking agents, in trigger and reverse triggering breaths. We hypothesized that the volume of pendelluft may be affected by respiratory mechanics and the radiological pattern of ARDS.

## Methods

### Study population

We selected ventilated patients who were over 18 years old and fulfilled Berlin’s diagnostic criteria for moderate to severe ARDS and received sedation and neuromuscular blocking agents to participate in our study. The exclusion criteria included patients with metallic materials in their bodies, cutaneous diseases that prohibited the application of electrode attachment to the body, severe chronic obstructive pulmonary diseases, unstable hemodynamic, proven barotrauma, pregnancy, diseases that included increased intracranial pressure, and surrogates who refused to provide informed consent (including esophageal balloon placement or EIT recording). The definition of unstable hemodynamics was the use of any vasopressor at a dose of ≥ 0.25 μg per kilogram of body weight per minute, sustained for at least 6 h, to maintain a systolic blood pressure of at least 90 mmHg or a mean blood pressure of 65 mmHg^8^. Studies on the pendelluft phenomenon in the ICU are rare. An observational study by Coppadoro et al. included a sample size of 20 cases^7^, while the study by Cornejo et al. recruited 24 cases^9^. No formal power calculation could be performed beforehand. However, we chose a sample size of 20 analyzable cases, which is consistent with previous studies on the pendelluft phenomenon. In our study, breaths for analysis should be free of artifacts. We defined sufficiently representative breaths as comprising 20% of all breaths, evenly spaced over the range of recorded respiratory effort.

### Study protocol

All selected patients who fulfilled the diagnosis of ARDS received volume-controlled ventilation under a constant flow. All patients were under sedation and paralysis. The criteria for interrupting the use of neuromuscular blocking agents were as follows: PaO_2_/FiO_2_ > 100 with FiO_2_ ≤ 0.6 and improved hemodynamics with the use of no more than one inotropic agent, dosed at < 0.25 μg per kilogram of body weight per minute, and maintaining a systolic pressure of at least 90 mmHg or a mean blood pressure of 65 mmHg. The neuromuscular blocking agent was reinstituted if one of the following criteria was met: 1) significant breathing effort with P_0.1_ > 6 cmH_2_O or onset of discomfort; 2) altered hemodynamics, including an increase in blood pressure (> 30 mmHg) or heart rate (> 30/min), or hypotension; 3) a need to increase FiO_2_ > 0.6. After discontinuing the use of neuromuscular blocking agents in these patients, we performed recordings at the transition from controlled to spontaneous ventilation. The recording was performed once for registered cases and recording durations of up to 60 min were allowed with minor adjustments according to the recording status (Supplementary Table 1E). The neuromuscular blocking agent was reinstituted earlier if the above-mentioned criteria were fulfilled. Mandatory ventilatory rates could be changed during the recording to promote the appearance of breathing efforts. Some reverse triggering breaths could be abolished by decreasing the mandatory breaths, according to our previous study results^10^. Recording time refers to the time from discontinuing the neuromuscular blocking agent to the end of recording, while count time refers to the time from steady active breathing started to be counted until the end of recording.

#### Respiratory and electrical impedance tomography measurement 

Air flow was measured in all patients using a pneumotachograph (PN 155,362, Hamilton Medical, Bonaduz, Switzerland) and differential pressure transducers (P/N 113,252, Model 1110A, Hans Rudolph, Shawnee, KS, USA). The flow sensor was placed between the endotracheal tube and the Y-piece of the ventilator, and tidal volume was obtained by integrating the flow signal. For each patient, an esophageal balloon (Cooper Surgical, Trumbull, CT) was positioned in the lower third of the esophagus to confirm an appropriate esophageal pressure signal according to standard guidelines^11^. Differential pressure transducers (P/N 113,252, Model 1110A, Hans Rudolph, Shawnee, KS, USA) were used to measure airway pressure and esophageal pressure. All signals were sampled and digitalized at 200 Hz and stored in a data acquisition system (MP150, AcqKnowledge, Biopac, Goleta, CA). For combined analysis with EIT data, pressure and flow signals were resampled to 20 Hz. We used a commercial EIT monitor (PulmoVista 500, Drager Medical GmbH, Lubeck, Germany) to display functional EIT images, which included relative impedance changes such as regional tidal ventilation and end-expiratory lung impedance (EELI) changes. EIT data were registered at 20 Hz and were low-pass filtered (40 per minute) during the study and stored for offline analysis. We subdivided the impedance recordings into four regions of interest (ROIs)^12^, from ventral to dorsal. The nadir of the global impedance of each breath was considered the transition from expiration to inspiration. The nadir of the impedance of the four ROIs could occur before, in time with, or after the global nadir. Pendelluft was defined as the absolute difference in impedance between the regional nadir and regional impedance at the time of the global nadir, and it was the sum of regional pendelluft values of the four ROIs^7,13^.

#### Definition of various breaths

##### Trigger breath

Decreases in P_es_ greater than 1 cm H_2_O were observed with subsequent ventilator-delivered breaths. Artifacts caused by cardiac oscillation or esophageal contraction were excluded from the analysis.

##### Reverse triggering breath

Defined as an inspiratory effort that occurs after a ventilator-initiated breath, without evidence of a patient-initiated assisted breath. Reverse triggering can be stable or unstable. Stable reverse triggering occurs in rhythmic patterns that are presumed to be secondary to respiratory entrainment. Unstable reverse triggering results from an uncoupled underlying oscillator, which occurs independently from the ventilator cycle^14^. Reverse triggering with breath stacking is classified as breathing stacking breaths.

##### Ineffective triggering during expiratory phase

A decrease in P_es_ greater than 1 cm H_2_O with a simultaneous drop in P_aw_ and/or changes in flow without subsequent ventilator-delivered breaths were observed, and artifacts caused by cardiac oscillation or esophageal contraction were excluded.

##### Breath stacking

This is defined as two cycles separated by a very short expiratory time, which is less than one-half of the mean inspiratory time. The first cycle can be patient-triggered or ventilator-delivered (i.e., reverse triggering).

#### Respiratory mechanics (Fig. 1) 

**Figure 1:**
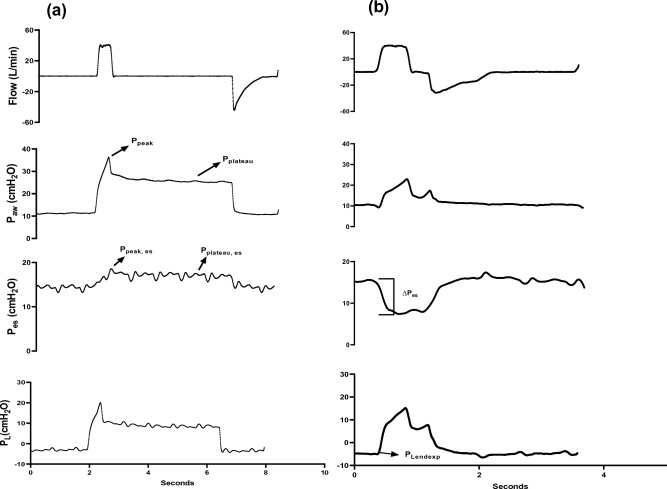
Schematic depiction of (a) Flow, P_aw_, P_es_ and P_L_ during Interrupter mechanics under constant flow ventilation (b) ∆P_es_ and P_Lendexp_ recordings during trigger breath. P_aw_, airway pressure; P_es_, esophageal pressure; P_peak_, peak airway pressure. P_Plateau_, airway plateau pressure. P_peak, es_, peak esophageal pressure. P_plateau, es_, plateau esophageal pressure. ∆P_es_, esophageal pressure swing; P_L_, transpulmonary pressure; P_Lendexp_, end-expiratory P_L_.

Total respiratory system, lung, and chest wall mechanics at rest: The interrupter method was used to measure respiratory mechanics under constant flow ventilation during paralysis^15^. Measurements were taken over three breaths, and the calculated values were averaged. Respiratory system elastance (E_rs_) was calculated as (airway plateau pressure−total PEEP) / tidal volume. Chest wall elastance (E_cw_) was calculated as (esophageal plateau pressure−end-expiratory esophageal pressure) / tidal volume. Lung elastance (E_L_) was calculated as E_rs_−E_cw_. Respiratory system resistance (R_rs_) was calculated as (peak airway pressure−plateau pressure) / flow, and chest wall resistance (R_cw_) was calculated as (peak esophageal pressure−esophageal plateau pressure) / flow. Plateau pressure was measured 3 s after initiating the inspiratory pause (no flow after volume insufflation), during which all measurements of P_aw_, P_es_ and their derivatives were acquired. Esophageal pressure swing (∆P_es_): This is the difference between peak and trough esophageal pressure in each spontaneous breath. Four classes of breathing effort were defined according to ∆P_es_. Rest refers to no breathing effort after termination of neuromuscular blocking agent use. Low effort is defined as ∆P_es_ < 5 cmH_2_O. Moderate effort is defined as 5 ≤ ∆P_es_ < 10 cmH_2_O. High effort is defined as ∆P_es_ ≥ 10 cmH_2_O. End-expiratory transpulmonary pressure (P_Lendexp_): This is the transpulmonary pressure (P_L_) just before the subsequent breath. ∆P_es_ was 0 cmH_2_O during controlled breaths. Absolute ∆P_es_ increased with progressively increasing breathing efforts. The exact locations of P_Lendexp_ were determined using airflow signals. The point of continuous positive airflow indicated the start of inspiration, and continuous negative airflow indicated expiration. P_Lendexp_ was reliably located just prior to the inspiratory phase.

#### Quantitative EIT measurement

##### Ventilation distribution

**Figure 2:**
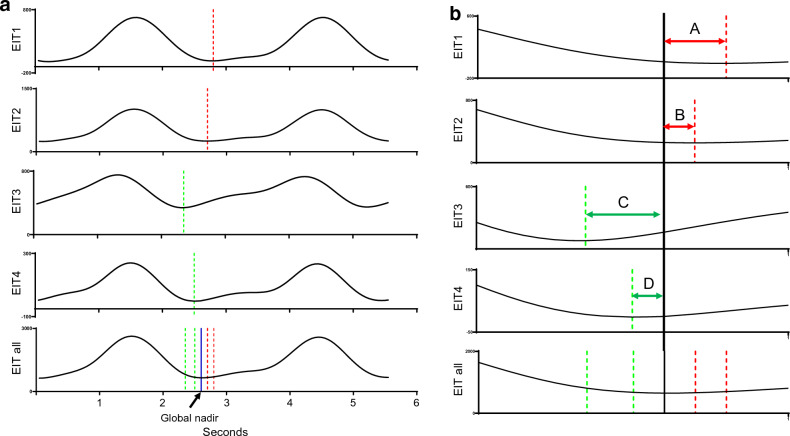
(**a**,**b**) Schematic representation of pendelluft measurement. Dotted lines refer to the location of regional nadir and solid lines refer to the location of global nadir. EIT1, ventral; EIT2, mid-ventral; EIT3, mid-dorsal; EIT4, dorsal. EIT all, total EIT. Pendelluft = A + B + C + D.

The two contiguous regions for the relative distribution of tidal ventilation were the gravitationally nondependent ROI, from halfway to the top of the imaging field, and the dependent ROI, from halfway ventilation to the bottom of the imaging field. Quantification of pendelluft (Fig. 2a,b): Pendelluft was quantified using the method described by Coppadoro et al. and the algorithm mentioned earlier^7^. Assessment of EELI: EELI was determined by referring to the nadir of the global impedance of each breath and served as a surrogate for end-expiratory lung volume (EELV)^12^.

#### Radiological classification

Bedside chest radiography taken at the time of recording was used to classify the radiological pattern of our patients. The chest X-ray findings were categorized into two patterns: involvement of the lung in two quadrants or fewer and involvement of the lung in more than two quadrants.

### Statistical analysis

The results were presented as mean ± SD values. Ventilatory parameters, such as tidal volume, duty cycle, P_Lendexp_, ∆P_es_, and EIT parameters (including ventilation distribution, EELI, and pendelluft), were recorded as primary endpoints for selected resting breathings, trigger breaths, and reverse triggering breaths under various levels of breathing effort. It should be noted that the numbers of trigger and reverse triggering breaths were not equal in different pressure ranges for each respective patient, and in some cases, certain pressure ranges were missing. To account for different ranges and numbers of breaths, the analysis of ventilatory and EIT parameters between rest and different levels of breathing effort utilized repeated measures ANOVA with generalized linear mixed models. Tukey’s multiple comparison test was employed to compare different efforts. Additionally, to examine the relationship between pendelluft and breathing effort, a simple linear regression was performed between pendelluft volume and ∆P_es_ for each patient. When the dependent variable was ordinal or continuous, Mann–Whitney U tests were used to compare between two independent groups. The Chi-squared test was applied for the analysis of a contingency table with categorical variables. A *p* < 0.05 was considered significant. Prism version 8 (GraphPad Software, San Diego, CA) and IBM SPSS Statistics Version 17 were utilized for the analysis.

### Ethics approval and consent to participate

This study was reviewed and approved by The Institutional Review Board of National Cheng Kung University Hospital (B-BR-107-082) and informed consent was obtained from patient’s legal representative according to Taiwan regulations. We conducted this study in accordance with good clinical practice guidelines and the Declaration of Helsinki.

## Results

**Figure 3:**
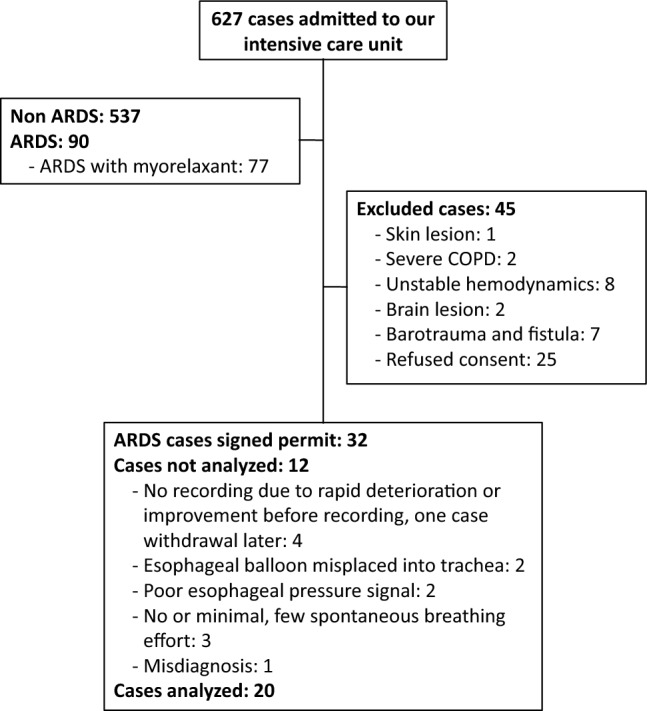
Screening and study flow diagram for patients with ARDS.

From December 1, 2019 to May 1, 2021, 32 patients with ARDS who met the criteria for a diagnosis of moderate to severe ARDS participated in the study (Fig. 3). Recordings were not further analyzed in twelve cases for various reasons (Fig. 3). A total of 20 recordings were available for analysis (Tables 1 and 2). All patients were under fentanyl and midazolam or propofol. All patients were in deep sedation (Richmond Agitation-Sedation Scale, − 4 to − 5). Seven patients were still under one inotropic agent. The average recording time was about 51.2 min, and the average count time was 27.5 min. Trigger breaths were recorded in all 20 cases. The most frequently observed asynchrony was reverse triggering, which was present in 18 recorded cases (2 cases were not further analyzed because only 4 and 7 reverse triggering breaths were recorded, respectively). It was continuously present or mixed with trigger or controlled breaths (i.e., stable or unstable reverse triggering, respectively). Breath stacking was present in 10 recorded cases and occurred sporadically in most of them and following reverse triggering. Ineffective triggering in the expiratory phase was present in six recorded cases; esophageal pressure monitoring data indicated that most cases of ineffective triggering were weak efforts. The recording results indicated that trigger and reverse triggering breaths were the most frequently occurring breath types. The number of trigger and reverse triggering breaths counted and analyzed is presented in Table 2. A total of 5155 trigger breaths and 3518 reverse triggering breaths were recorded. For the analysis, we randomly selected 20% of the qualified recorded trigger and reverse triggering breaths following the classification of breathing effort as low, moderate, and high effort according to ∆P_es_ results. In all patients, 10 rest breaths (∆P_es_ = 0 cmH_2_O) were selected. For trigger and reverse triggering breaths, a minimum of 10 breaths were selected if the number of breaths within that pressure range was less than 50. In cases of limited breaths (< 10) in a respective effort range, we selected all of them. In total, 200 resting breaths, 1135 trigger breaths, and 807 reverse triggering breaths were selected and analyzed (Table 2). All breath data were acquired during volume-cycled, constant flow ventilation. The results for respiratory parameters and pendelluft at baseline and during trigger and reverse triggering breaths are presented in Table 3. ∆P_es_ and pendelluft were significantly higher in trigger breaths than in reverse triggering breaths. P_Lendexp_ was significantly lower in trigger breaths than reverse triggering breaths too. Additional information, including breath stacking and ineffective triggering, is provided in Supplementary Table 1E.

**Table 1:** Patient characteristics and respiratory mechanics.

Case	Age/ Gender	Cause of ARDS (CXR pattern)#	Day on MV	FiO_2_/PEEP	Tidal volume (ml/IBW in Kg)	P/F ratio	R_rs_ (cmH_2_O/L/S)	E_L_/E_cw_ (cmH_2_O/L)	∆P_es_/∆P_aw_
1	86/F	Cholangiocarcinoma with pneumonia (2)	7	0.4/10 cmH_2_O	6.1 ml/Kg	197.5	24.6	27.3/6.8	0.9
2	54/M	ANCA GN with PJP (2)	8	0.5/12 cmH_2_O,	5.9 ml/Kg	122	14.9	22.0/4.5	0.9
3	47/F	Liver abscess with pneumonia (2)	8	ECMO, 0.5/13 cmH_2_O	6.1 ml/ Kg		20.9	31.1/8.3	0.8
4	43/M	Rectal cancer with VP (2)	7	0.35/10 cmH_2_O	6.0 ml/Kg	220	10.0	14.8/2.3	1.0
5	68/M	RP (1)	3	0.4/10 cmH_2_O	6.1 ml/Kg	202.5	14.6	17.0/5.2	1.1
6	79/F	PJ (2)	4	0.5/10 cmH_2_O,	7.8 ml/Kg	142	16.0	49.2/5.3	1.1
7	92/M	VP (2)	2	0.4/10 cmH_2_O	6.1 ml/Kg	230	16.4	35.5/6.7	1.1
8	48/M	Ankylosing spondylitis with VP (2)	2	0.45/10 cmH_2_O	6.7 ml/Kg	178	14.7	18.6/4.4	1.0
9	60/M	Pneumonia (1)	2	0.45/10 cmH_2_O	6.6 ml/Kg	160	17.5	28.7/3.7	1.1
10	43/M	Antisynthetase syndrome with pneumonia (2)	12	0.4/8 cmH_2_O	6.6 ml/Kg	195	13.1	32.6/5.0	0.8
11	73/F	Polymicrobial bacteremia (1)	5	0.4/8 cmH_2_O	6.5 ml/Kg	188	13.4	40/11.4	0.9
12	66/M	Lung cancer with PJP (2)	16	0.4/10 cmH_2_	6.3 ml/Kg	145	15.2	31.9/7.0	1.1
13	70/M	Hypopharyngeal cancer with PJP (1)	3	0.4/10 cmH_2_O,	6.9 ml/Kg change to 8.0 ml /Kg when recording	138	14.1	16.4/9.2	1.0
14	36/F	SLE with VP (2)	2	0.5/10 cmH_2_O	6.9 ml/Kg	172	15.7	35.2/5.8	1.0
15	86/M	OP (F)	6	0.45/12 cmH_2_O	7.1 ml/Kg	171	16.9	19.2/8.3	0.9
16	74/M	Dermatomyositis with VP (1)	15	0.5/12 cmH_2_O	6.6 ml/Kg	168	17.3	19.5/10.9	1.1
17	88/M	Pneumonia (1)	2	0.4/8 cmH_2_O	6.2 ml/Kg	183	18.8	14.0/11.1	0.9
18	59/M	Pneumonia (1)	14	0.4/12 cmH_2_O,	6.3 ml/Kg	218	10.9	20.8/2.6	1.1
19	72/M	Pancreatic cancer with polymicrobial bacteremia(1)	10	0.4/12 cmH_2_O,	6.6 ml/Kg	170	15.8	14.4/4.9	1.0
20	72/F	MRSA bacteremia (2)	3	0.4/10 cmH_2_O	6.5 ml/Kg	188	16.7	38.7/8.9	0.9

M, male; F, female; CXR, chest X-ray; ∆P_es_/∆P_aw_, esophageal pressure and airway pressure swing ratio during chest compression test; MV, mechanical ventilation; R_rs_, respiratory system resistance in in volume cycle constant flow ventilation; E_L_, lung elastance; E_cw_, chest wall elastance; ∆P_es_, esophageal pressure swing during active breath; ANCA, anti-neutrophil cytoplasmic antibody; GN, glomerulonephritis; ECMO, extracorporeal membrane oxygenation; OP, organizing pneumonia; PJP, Pneumocystis jirovecii pneumonia; RP, radiation pneumonitis; SLE, Systemic Lupus Erythematosus; VP, viral pneumonia; IBW, ideal body weight; MRSA, methicillin-resistant staphylococcus aureus; P/F ratio, PaO_2_ to FiO_2_ ratio.

^#^Radiological pattern: 1: involvement of ≤ 2 quadrants of lung. 2: involvement > 2 quadrants of lung.

**Table 2:** Number of trigger and reverse triggering breaths^#^ analyzed/count^##^ and patient outcome.

Case	Trigger breaths (analyzed/count)	Trigger breaths ∆P_es_ (cmH_2_O) range	Reverse triggering breaths (analyzed/count)	Reverse triggering breaths ∆P_es_ (cmH_2_O) range	Outcome
1	146/707	3.3–21.7			D
2	23/82	3.1–10	56/278	3.3–8.5	D
3	200/1000	2–15.9			S
4	20/30	7.3–13.8	131/635	2–12.9	S
5	24/75	4.1–31	39/194	3.1–8.2	D
6	10/15	14–15	108/533	3.2–14.7	D
7	59/288	2–13.1	26/65	3–16.5	D
8	19/56	5.8–14.1	64/297	2.5–13.1	S
9	43/208	7.1–11.4	20/24	7.6–12.2	S
10	101/500	2.6–26.3			S
11	20/24	5–15.5	55/265	2.7–19.9	D
12	36/146	2.5–14.8	45/202	2.7–14.1	D
13	32/162	1.4–9.9	24/89	2.9–10.9	D
14	138/681	2–13.7			S
15	21/61	3–18.1	42/176	2.8–13.1	D
16	20/41	7.3–10.9	57/240	3.3–10.5	D
17	28/140	2.6–9.8	20/46	2.6–6.5	S
18	54/266	2.1–16.7	21/74	2.3–6.7	S
19	98/488	2.3–13.7	44/172	3.9–14.2	D
20	43/185	4–16.7	55/227	3.7–13.1	S

∆P_es_, esophageal pressure swing during active breath; S, survival; D, death.

^#^Scattered reverse triggering breaths less than 10 breaths in 2 case were not included for analysis.

^##^Count breaths refer to steady active breathing started to be counted until the end of recording.

**Table 3:** Respiratory parameters and pendelluft at rest and during trigger, reverse triggering breaths.

	Rest (n = 200)	Trigger breaths (n = 1135)	Reverse triggering breaths (n = 807)
Respiratory parameters			
Tidal volume (ml)	347.8 ± 48.9	360.3 ± 53.3*	363.6 ± 71.2*
Ti (second)	0.86 ± 0.09	0.94 ± 0.13**	0.95 ± 0.21**^##^
Te (second)	2.04 ± 0.57	2.18 ± 0.78*	2.29 ± 0.81** ^#^
End-expiratory P_L_ (P_Lendexp_) (cmH_2_O)	0.4 ± 3.0	− 1.5 ± 4.1**	− 0.5 ± 3.4* ^##^
∆P_es_ (cmH_2_O)	0.0	9.2 ± 4.6	6.9 ± 2.9 ^##^
Pendelluft (ml)	0.5 ± 0.8	3.9 ± 6.8**	1.9 ± 2.8** ^##^

Ti, inspiratory time; Te, expiratory time; * *p* < 0.05, **P < 0.0001 between trigger and rest breaths, between reverse triggering and rest breaths; # *p* < 0.05, ## *p* < 0.0001 between trigger and reverse triggering breaths.

### EIT measurement and P_Lendexp_ in trigger breaths (Fig. 4)

**Figure 4:**
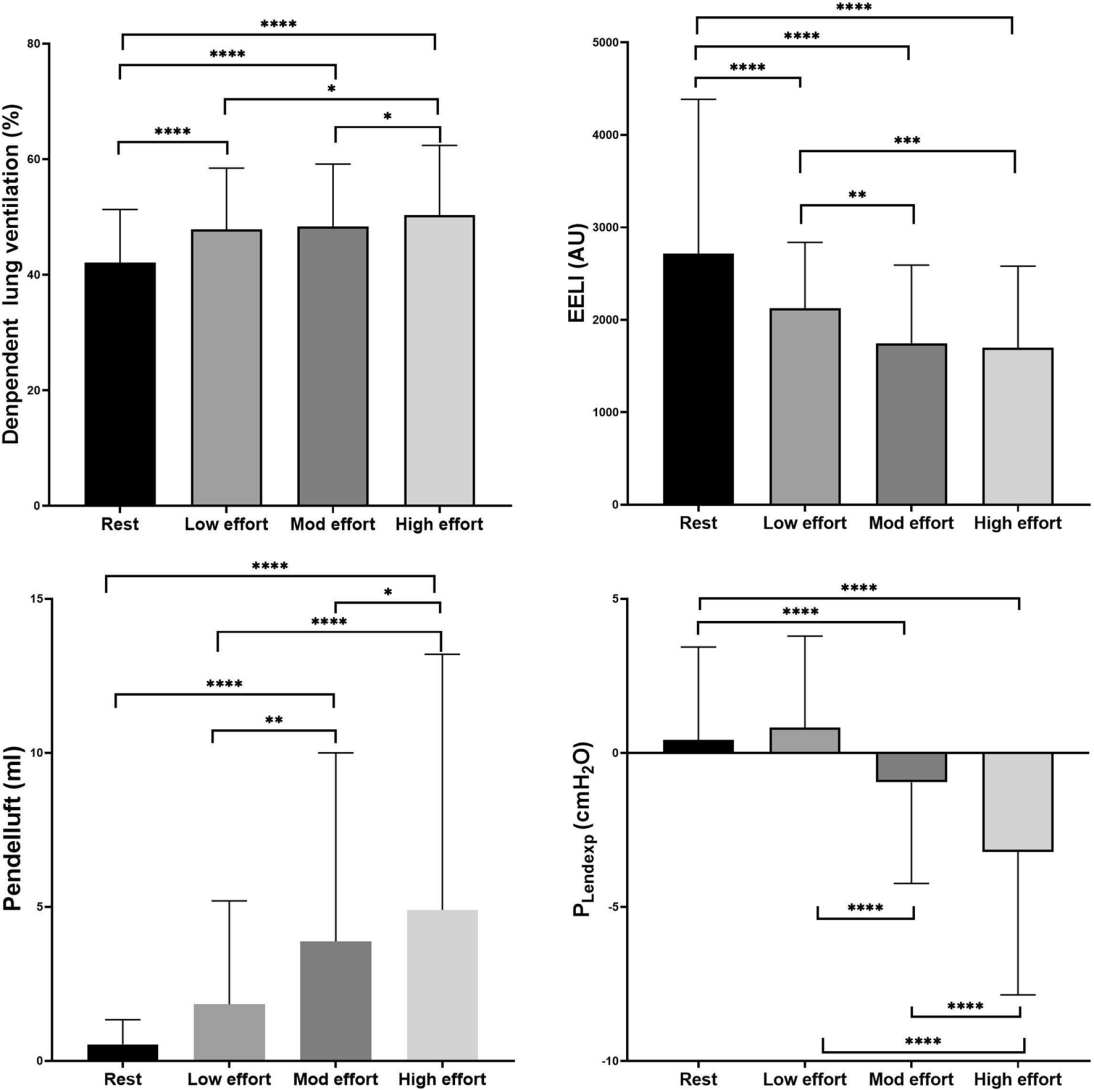
Ventilation distribution, pendelluft, EELI, P_Lendexp_ at rest and various levels of breathing effort during trigger breaths. EELI, end-expiratory lung impedance; P_Lendexp_, end-expiratory transpulmonary pressure; Mod effort, moderate effort. * *p* < 0.05; ** *p* < 0.01***; *p* < 0.001; **** *p* < 0.0001.

For trigger breaths, ∆P_es_ averaged 3.6 ± 0.8 cmH_2_O, 7.5 ± 1.5 cmH_2_O, and 13.9 ± 3.4 cmH_2_O for low (224 breaths), moderate (467 breaths), and high effort (444 breaths), respectively. Tidal ventilation distribution changed with the degree of breathing effort, and ventilation significantly increased in the dependent lung regions as effort increased. EELI changed during spontaneous breathing trials and was significantly decreased with increasing breathing effort. Pendelluft significantly increased with progressively greater breathing effort. The average P_Lendexp_ became progressively negative with moderate to high respiratory efforts.

### EIT measurement and P_Lendexp_ in reverse triggering breaths (Fig. 5)

**Figure 5:**
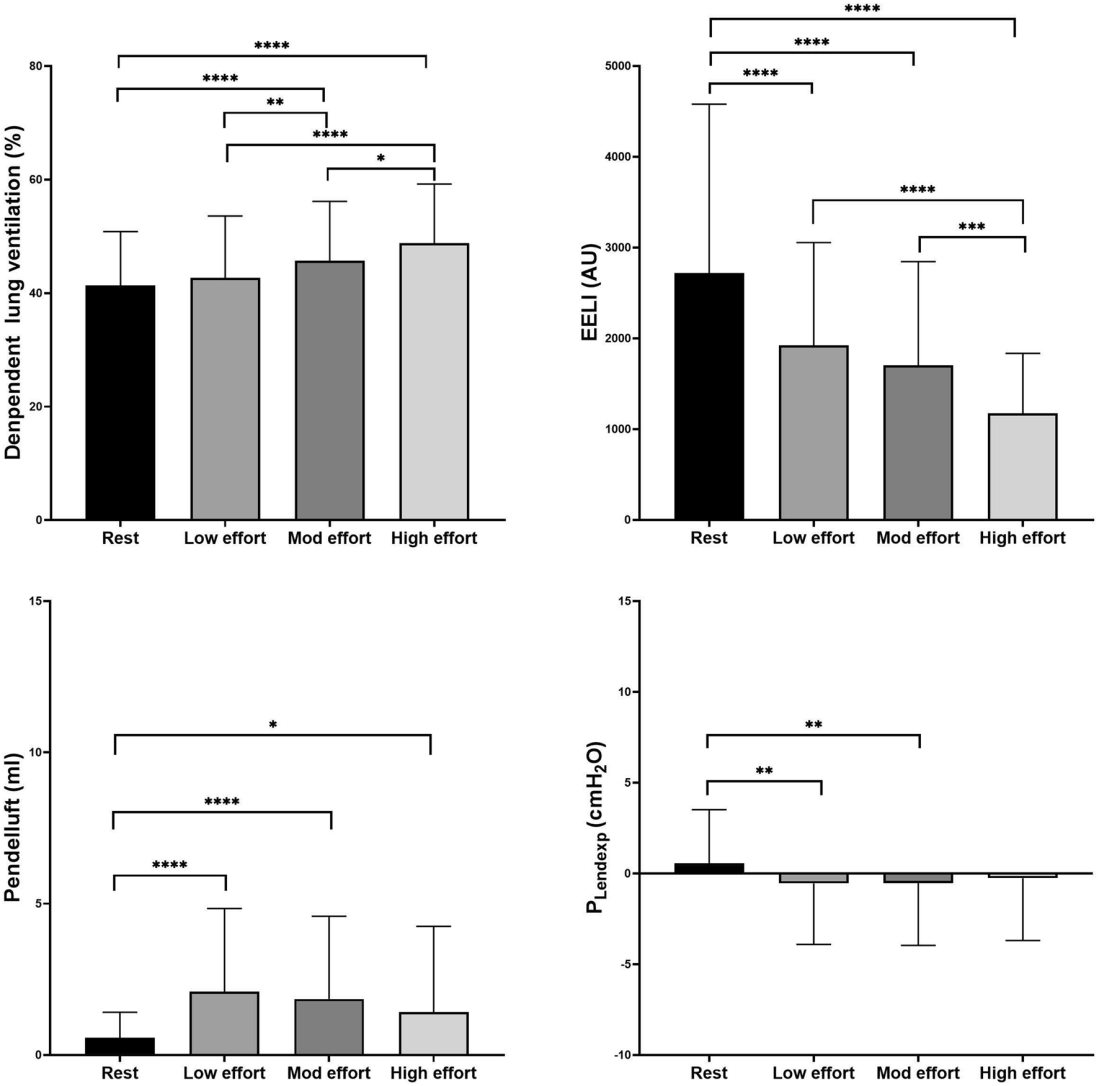
Ventilation distribution, pendelluft, EELI, P_Lendexp_ at rest and various levels of breathing effort during reverse triggering breaths. EELI: end-expiratory lung impedance; P_Lendexp_, end-expiratory transpulmonary pressure; Mod effort, moderate effort. * *p* < 0.05; ** *p* < 0.01***; *p* < 0.001; **** *p* < 0.0001.

For reverse triggering breaths, ∆P_es_ averaged 3.9 ± 0.7 cmH_2_O, 7.2 ± 1.4 cmH_2_O, and 12.0 ± 1.8 cmH_2_O for low (247 breaths), moderate (439 breaths), and high effort (121 breaths) breathing, respectively. Ventilation also significantly increased in dependent lung regions as effort increased during reverse triggering breaths. EELI changed during spontaneous breathing and was significantly decreased as breathing effort increased. Pendelluft increased with all breathing effort, and P_Lendexp_ was significantly decreased with efforts compared with at-rest breaths, but there were no differences between different levels of breathing effort.

### High and low pendelluft in trigger and reverse triggering breaths

Pendelluft occurred dissimilarly with increasing breathing effort in individual patients. Analysis of the slopes of the linear regression curves between ∆P_es_ and pendelluft revealed two distinct effort-related pendelluft patterns in trigger breaths (Table 4). We defined the high pendelluft group as pendelluft-∆P_es_ slope greater than or equal to 0.5 ml/ ml/cmH_2_O. For trigger breaths, the high pendelluft group was significantly associated with lower peak airway/plateau pressure and lower respiratory system /lung elastance. The focal radiological pattern was also predominant in the high pendelluft group. For reverse triggering breaths, the slope values for four patients were 0.5–2.6 ml/cmH_2_O (high pendelluft group), and the slope values for the other 12 patients were − 0.2 to 0.3 ml/cmH_2_O (low pendelluft group). For reverse triggering breaths, there was no difference in respiratory mechanics between high and low pendelluft groups. For trigger breaths, the pendelluft predicted by ΔPes is shown in the Table 5. The prediction of pendelluft by ∆P_es_ was characterized by a low positive predictive value. ARDS cases with high effort-related pendelluft were uncommon. The prediction of pendelluft was not significant in reverse triggering breaths. (The relationship among inspiratory muscle pressure (P_mus_), esophageal pressure time product (PTP_es_), and ∆P_es_ were shown in Fig. 1E and 2E, and the prediction of pendelluft in trigger breaths based on P_mus_ was shown in Supplementary Table 2E).

**Table 4:** Clinical characteristics and passive respiratory mechanics under paralysis between high and low pendelluft group during trigger breaths.

	High pendelluft group (n = 9)	Low pendelluft group (n = 11)
Pendelluft-∆P_es_ slope (ml/cmH_2_O)	1.2 ± 0.5 (0.7 to 2.4)	0.1 ± 0.1 (− 0.1 to 0.3)
Gender (M/F)	8/1	6/5
Age (years)	64 ± 14	67 ± 19
Flow (L/min)	40.3 ± 1.1	40.2 ± 4.9
Tidal volume (ml)	373.6 ± 49.3	346.0 ± 50.6
P_peak_ (cmH_2_O)	30.5 ± 3.2	36.5 ± 1.8*
P_pla_ (cmH_2_O)	20.8 ± 2.6	25.2 ± 2.0*
PEEP (cmH_2_O)	11.7 ± 1.4	11.9 ± 1.8
E_rs_ (cmH_2_O/L)	25.3 ± 9.1	39.0 ± 8.3*
E_cw_ (cmH_2_O/L)	5.9 ± 3.1	7.2 ± 2.4
E_L_ (cmH_2_O/L)	19.4 ± 7.9	31.8 ± 8.6*
R_rs_ (cmH_2_O/L/S)	14.5 ± 2.7	17.0 ± 3.3
R_cw_ (cmH_2_O/L/S)	0.7 ± 0.3	0.5 ± 0.2
Radiological pattern(1/2)^#^	6/3	2/9**

P_peak_, peak airway pressure; P_pla_, airway plateau pressure; E_rs_, respiratory system elastance; E_cw_: chest wall elastance; E_L_, lung elastance; R_rs_, respiratory system resistance, R_cw_, chest wall resistance. *p < 0.01 ** *p* < 0.05.

^#^Radiological pattern: 1: involvement of ≤ 2 quadrants of lung. 2: involvement > 2 quadrants of lung.

**Table 5:** Performance of ∆P_es_ in the prediction of pendelluft volume in triggered breaths (n = 1135).

Pendelluft (ml)	∆P_es_ threshold (cmH_2_O)	Sensitivity	Specificity	PPV	NPV	AUC	*P* value
5.0	6.75	0.78	0.38	0.28	0.84	0.576	*P* < 0.001
10.0	8.55	0.73	0.51	0.14	0.95	0.606	*P* = 0.002
15.0	8.65	0.78	0.51	0.09	0.97	0.649	*P* = 0.001
20.0	8.65	0.82	0.51	0.07	0.98	0.666	*P* < 0.001
25.0	8.65	0.84	0.51	0.05	0.99	0.676	*P* = 0.002

∆P_es_, esophageal pressure swing; PPV, positive predictive value. NPV, negative predictive value; AUC, area under curve.

## Discussion

Our study revealed that pendelluft occurred in patients with ARDS under volume-controlled ventilation when neuromuscular blocking agents were discontinued and active breathing started. We made several important findings. Firstly, the pendelluft response to breathing effort varied and could be practically divided into high and low pendelluft groups based on the slope of pendelluft-∆P_es_ relationship. Additionally, pendelluft volumes were significantly higher in trigger breaths compared to reverse triggering breaths. Secondly, ARDS patients with a high pendelluft response in trigger breaths were characterized by significantly lower lung elastance, lower airway pressure, and a focal radiological pattern. Thirdly, as EELI and P_Lendexp_ decreased with increasing breathing effort in both trigger and reverse triggering breaths, this implicated that reduced lung volume and atelectasis might be prone to occur with breathing effort. Lastly, predicting the amount of pendelluft in trigger breaths based on ∆P_es_ was characterized by a low positive predictive value and a high negative predictive value.

It is suggested that appropriate spontaneous breathing should be used to avoid diaphragm injury following the use of neuromuscular blocking agents in patients with ARDS^16,17^. During the transition from passive to active breathing following the termination of neuromuscular blocking agents, we found different patterns of patient-ventilator interactions. Some patients had low breathing effort, but more patients resumed trigger breaths of varying degrees of effort, and we found several types of patient-ventilator dyssynchrony. Reverse triggering with or without breath stacking was the predominant patient-ventilator dyssynchrony in our patients. Deep sedation is a possible factor for the occurrence of reversing triggering breaths^18^.

Air moves preferentially to ventral regions of the lung during paralyzed, controlled ventilation. However, the dependent part of the diaphragm moves the most during spontaneous breathing, resulting in increased ventilation to the dorsal lung region^19^. In patients with ARDS who undergo pressure support ventilation, a high breathing effort also leads to increased distribution of ventilation in the dependent lung regions compared with a low breathing effort^3^. Our patients were under volume-cycled ventilation, and an increased ventilation distribution in dependent lung regions also occurred as breathing effort increased. This result suggested that there were similar dependent diaphragm movements during trigger and reverse triggering breaths.

Pendelluft occurred in trigger and reverse triggering breaths as expected, and our study found that pendelluft was more pronounced during trigger breaths, but pendelluft volumes were divergent. Interestingly, ARDS patients with high pendelluft were characterized by low lung elastance and lower airway pressure, which may promote gas flow with breathing effort. From a chest CT study, it is clear that ARDS is characterized by varying levels of alveolar aeration and collapse. In this heterogeneous distribution, the lung can be viewed as focal solid areas that resist shape deformation and thus may cause imperfect elastic anisotropic inflation and facilitate pendelluft^4^. It is known that focal ARDS is associated with lower lung elastance^20^, which may explain our findings. However, predicting pendelluft severity based on breathing effort during trigger breaths was characterized by a high negative predictive value and a low positive predictive value.

P_Lendexp_ is a surrogate for tidal alveolar derecruitment and is used to guide ARDS ventilator strategy^21^. P_Lendexp_ decreased with breathing effort in both trigger and reverse triggering breaths. There are several explanations for the decreased P_Lendexp_, but decreased lung volume may be the main reason for the decrease in P_Lendexp_^22^. Evidence supporting our reasoning comes from the EELI measurement results, which show that EELI decreased significantly with increasing ∆P_es_. Another explanation for decreased P_Lendexp_ is the activation of expiratory respiratory muscle following the discontinuance of neuromuscular blocking agents. High respiratory drive can activate expiratory muscles, leading to elevated end-expiratory pleural pressures and decreased P_Lendexp_ values^23,24^. Relaxation of inspiratory muscles during trigger breaths is similar to expiratory muscle activation, which also decreases P_Lendexp_ values^25^.

However, this study had some limitations. First, the choice of breaths examined might have led to selection bias and imbalance. We analyzed 20% of qualified recorded trigger and reverse triggering breaths, so we could not directly extend our results to the unanalyzed breaths. However, we chose the breaths based on the ∆Pes range to ensure that we gathered a sufficient number of representative breaths for analysis. Additionally, we used a statistical method to address the inherent limitation of some imbalance in the distribution of pressure ranges in individual cases. Second, all patients analyzed underwent volume-controlled, constant flow ventilation. Thus, our results might not apply to different modes of ventilation. Third, to estimate pendelluft volume, we used the method developed by Coppadoro et al.^7^, which assumes that four homogeneous lung zones are present. However, this assumption can lead to underestimation of pendelluft volume^7^. Nonetheless, the method used was physiologically sound. Fourth, this was a single-center study, and further validation at other institutes may be needed.

In summary, trigger and reverse triggering breaths in patients with ARDS resulted in significant changes in P_Lendexp_ and regional ventilation distribution. Both trigger and reverse triggering breaths led to negative P_Lendexp_. Effort-related pendelluft volume was variable, and high pendelluft is prone to occur in patient with lower lung elastance during trigger breaths. High pendelluft during active breathing in patients with ARDS is uncommon in our study. Different ventilatory strategies may be applied in patients with ARDS with different effort-related pendelluft responses.

### Supplementary Information

Supplementary Figure 1.

Supplementary Figure 2.

Supplementary Information 1.

Supplementary Table 1.

Supplementary Table 2.

## Data Availability

The dataset used during the current study may be available from the corresponding author on reasonable request.
