# Supplementary figures and images for: Pendelluft in patients with acute respiratory distress syndrome during trigger and reverse triggering breaths
Source: Sci Rep. 2023 Dec 13;13:22143. doi: 10.1038/s41598-023-49038-9 (PMC10719360; doi:10.1038/s41598-023-49038-9)

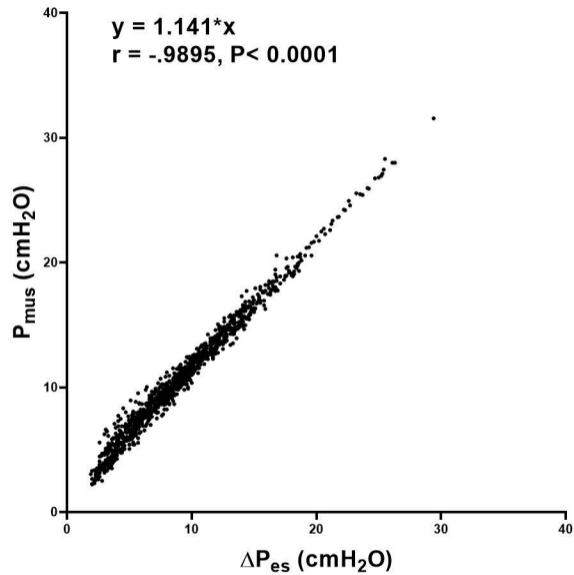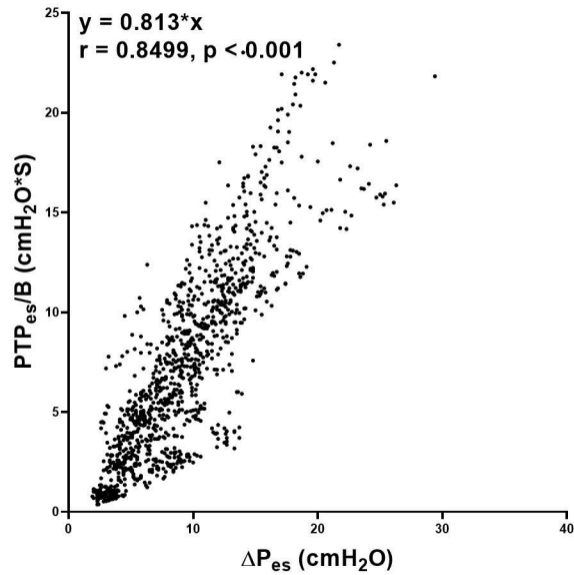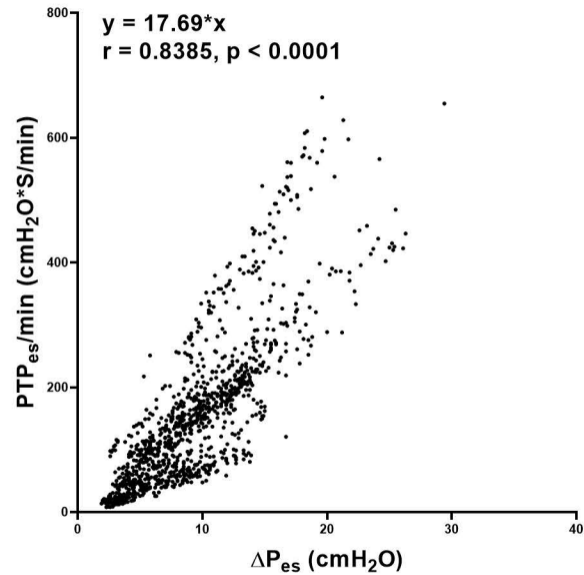

Supplement: Supplementary file 1 — Supplementary Figure 1. [file 41598_2023_49038_MOESM1_ESM.pdf]

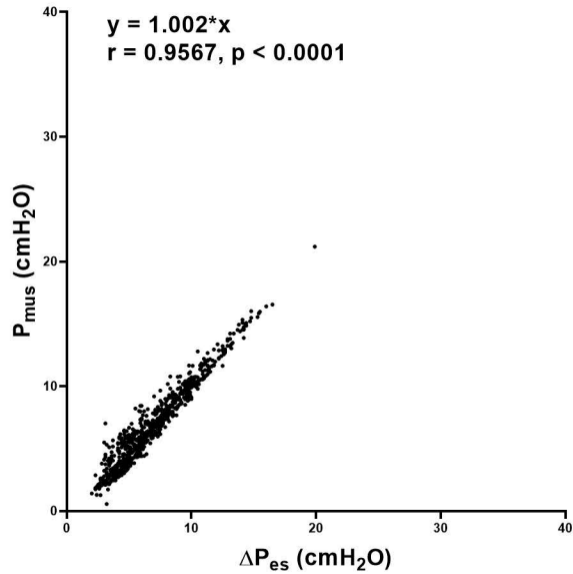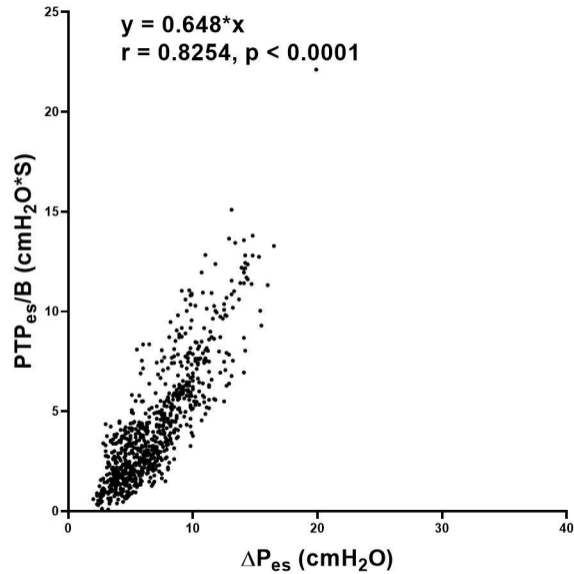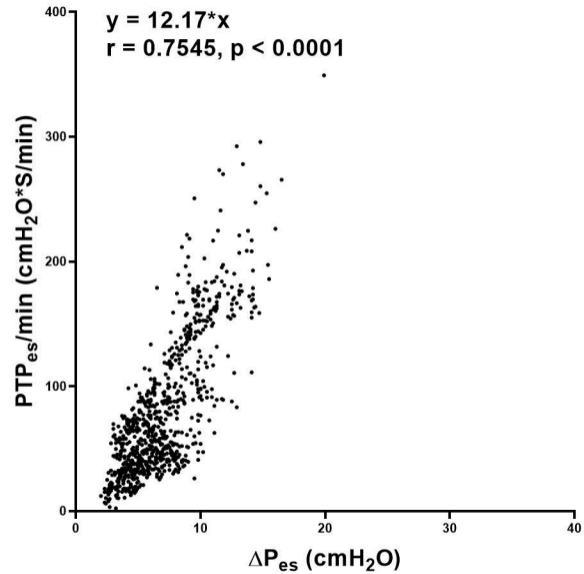

Supplement: Supplementary file 2 — Supplementary Figure 2. [file 41598_2023_49038_MOESM2_ESM.pdf]
