# Supplementary material for: Pendelluft in patients with acute respiratory distress syndrome during trigger and reverse triggering breaths
Source: Sci Rep. 2023 Dec 13;13:22143. doi: 10.1038/s41598-023-49038-9 (PMC10719360; doi:10.1038/s41598-023-49038-9)
Supplement: Supplementary file 3 — Supplementary Information 1. [file 41598_2023_49038_MOESM3_ESM.docx]

**Pendelluft in Patients with Acute Respiratory Distress Syndrome during trigger and reverse triggering breaths**

**—Supplemental Digital Content**

Wei-Chieh Lin, M.D., Ph.D.*, Pei-Fan Su, Ph.D.**, Chang-Wen Chen, M.D., M.S. *

*Section of Critical Care Medicine, Department of Internal Medicine, National Cheng Kung University Hospital, College of Medicine, National Cheng-Kung University, Tainan, Taiwan

** Department of Statistics, National Cheng Kung University, Tainan, Taiwan

Correspondence: [cwchen@mail.ncku.edu.tw](mailto:cwchen@mail.ncku.edu.tw)

Chang-Wen Chen, M.D., M.S. Section of Critical Care Medicine, Department of Internal Medicine, National Cheng Kung University Hospital, College of Medicine, National Cheng-Kung University, Tainan, Taiwan

**Research Support**

This study was supported by the Ministry of Science and Technology grant

MOST 108-2314-B-006-069.

**Competing Interests**

The authors declare no competing interests.

**Recording and selection of active breaths**

Timing of active breath counts varied with different patients. Usually occurring 10 to 20 minutes after discontinuation of myorelaxants, active breaths were at first generally sporadic and weak. In some cases, the active breaths occurred earlier or later. No active breaths occurred in one case. We started to count active breaths as steady active breaths were observed and confirmed. The results for durations of counted breaths are presented in Table 1E. With a progressive increase in breathing effort by the end of the recording, the ventilator mode sometimes changed to pressure control ventilation to alleviate the effort. However, this intervention was not always effective, and reinstitution of myorelaxant was necessary. Breaths during pressure control ventilation that was performed in very short intervals (1 to 4 minutes when the patient had significantly increasing effort) were not analyzed, because the study aimed at volume control ventilation and myorelaxant use was always re-implemented. No further recordings were performed thereafter. No changes of PEEP during recording.

**Estimation of inspiratory muscle pressure (P_mus_), esophageal pressure time product (PTP_es_), and ∆P_es_**

P_mus_ generated by the respiratory muscles were calculated with consideration of both the elastic and resistive properties of the chest wall[1]. The passive esophageal pressure was calculated with the motion equation: Passive P_es_=volume* E_cw_+flow*R_cw_ + end-expiratory P_cw_. The PTP_es_ per breath (PTP_es_/B) was obtained by calculating the area subtended by active P_es_ and passive P_es_ following superimposition the two curves. We only calculate the inspiratory portion. Inspiratory PTP_es_ per min (PTP_es_/min) was obtained by dividing the PTP_es_/B by breath duration. Because volume-controlled constant flow ventilation was used, the start of inspiratory effort was assumed to be the moment of rapid P_es_ decay; the end of inspiratory effort was assumed to be the P_es_ that elapsed 25% of the time from its maximum deflection to return to baseline[2]. For trigger breaths, the result for the correlation between ∆P_es_ and P_mus_, PTP_es_ is presented in Figure 1E. For reverse triggering breaths, the result for the correlation between ∆P_es_ and P_mus_, PTP_es_ was presented in Figure 2E. Linear regression analysis was performed between pendelluft volume and ∆P_es_, P_mus_, PTP_es_/B, and PTP_es_/min. Prediction of pendelluft in trigger breath based on P_mus_ was shown in Table 2E.

**Phenotypes of reverse triggering breaths**.

According to Kassis et al[3], phenotypes can be classified in reverse triggering breaths. In contrast to Kassis’s finding, tidal volume was minimally increased in our study because volume control ventilation was used. We identified four types of reverse triggering breaths according to Kassis’s proposal. Namely, early reverse triggering with early relaxation (A), early reverse triggering with delayed relaxation (B), mid-cycle reverse triggering with maximal inspiratory effort during lung inflation (C), and late reverse triggering with reverse triggering occurred completely during lung deflation (D). In our study, the selected 807 reverse triggering breaths revealed a distribution of 15 type A, 105 type B, 622 type C, and 65 type D. The phase delay averaged 0.5 + 0.2 second. Phase angle averaged 60.0 + 28.6 degrees. As type C reverse triggering breaths predominated, we did not further analyze the differences between these reverse triggering phenotypes.

**Legends**

**Figure 1E.** Correlation analysis between P_mus_, PIP_es_/B, PTP_es_/min vs ∆P_es_ in trigger breaths. P_mus_: inspiratory muscular pressure. PTP_es_/B: esophageal pressure time product per breath. PTP_es_/min: esophageal pressure time product per minute. ∆P_es_: esophageal pressure swing.

**Figure 2E.** Correlation analysis between P_mus_, PIP_es_/B, PTP_es_/min vs ∆P_es_ in reverse triggering breaths. P_mus_: inspiratory muscular pressure. PTP_es_/B: esophageal pressure time product per breath. PTP_es_/min: esophageal pressure time product per minute. ∆P_es_: esophageal pressure swing.

**References**

1. Kondili E, Alexopoulou C, Xirouchaki N, Vaporidi K, Georgopoulos D: **Estimation of inspiratory muscle pressure in critically ill patients**. *Intensive Care Med* 2010, **36**(4):648-655.

2. Damiani LF, Engelberts D, Bastia L, Osada K, Katira BH, Otulakowski G, Goligher EC, Reid WD, Dubo S, Bruhn A *et al*: **Impact of Reverse Triggering Dyssynchrony during Lung-Protective Ventilation on Diaphragm Function: An Experimental Model**. *Am J Respir Crit Care Med* 2022, **205**(6):663-673.

3. Baedorf Kassis E, Su HK, Graham AR, Novack V, Loring SH, Talmor DS: **Reverse Trigger Phenotypes in Acute Respiratory Distress Syndrome**. *Am J Respir Crit Care Med* 2021, **203**(1):67-77.
