# Supplementary material for: Pendelluft in patients with acute respiratory distress syndrome during trigger and reverse triggering breaths
Source: Sci Rep. 2023 Dec 13;13:22143. doi: 10.1038/s41598-023-49038-9 (PMC10719360; doi:10.1038/s41598-023-49038-9)
Supplement: Supplementary file 4 — Supplementary Table 1. [file 41598_2023_49038_MOESM4_ESM.docx]

**Table 1E. Record and count duration, pressure range of trigger breath, reverse triggering breath, breath stacking and ineffective triggering in expiratory phase**

| **Case** | **Record time**  **(seconds)**  **Count time**  **(seconds)** | **Trigger breaths (breaths analyzed/count, ∆P_es_ range in analyzed breaths/∆P_es_ range in count breaths (cmH_2_O))** | | **Reverse triggering breaths (breaths analyzed/count, ∆P_es_ range in analyzed breaths/∆P_es_ range in count breaths (cmH_2_O))** | **Breath stacking (breath count, ∆P_es_ range (cmH_2_O))** | **Ineffective triggering in expiratory phase (breath count , ∆P_es_ range (cmH_2_O) )** |
| --- | --- | --- | --- | --- | --- | --- |
| 1 | **(2290)**  **(2012)** | **(146/707, 3.3-21.7/2.8-21.7)** | | **7 breath (6.1-7.8 cmH_2_O) —not analyzed** |  | **97, 3.2-11.2** |
| 2 | **(2566)**  **(1576)** | **(23/82, 3.1-10/3.1-10)** | | **(56/278, 3.3-8.5/ 1.4-8.8)** |  |  |
| 3 | **(4180)**  **(4002)** | **(200/1000, 2-15.9/1-15.9)** | | **4 breaths (3.4-7.3 cmH_2_O)—not analyzed** |  | **33, 1.3-3.4** |
| 4 | **(3606)**  **(3147)** | **(20/30, 7.3-13.8/7.7-13.8)** | | **(131/635, 2-12.9/1.9-13.8)** | **62, 5.1-13.8** |  |
| 5 | **(2751)**  **(1364)** | **(24/75, 4.1-31/4.1-31)** | | **(39/194, 3.1-8.2/3.1-9.2)** | **61, 6.3-10.4** |  |
| 6 | **(2967)**  **(1602)** | **(10/15, 14-15/14-15)** | | **(108/533, 3.2-14.7/3.1-14.9)** |  |  |
| 7 | **(2411)**  **(1570)** | **(59/288, 2-13.1/1.3-16.3)** | | **(26/65, 3-16.5/3-17.8)** | **6, 15.8-18.7** | **42, 1.1-2.2** |
| 8 | **(2525)**  **(1353)** | **(19/56, 5.8-14.1/5.8-14.1)** | | **(64/297, 2.5-13.1/1.6-13.1)** | **2, 12.4-13.8** |  |
| 9 | **(1742)**  **(821)** | **(43/208, 7.1-11.4/6.1-14.5)** | | **(20/24, 7.6-12.2/7.6-12.2)** | **24, 9.5-14.4** |  |
| 10 | **(3031)**  **(1712)** | **(101/500, 2.6-26.3/2.6-27.9)** | |  |  |  |
| 11 | **(3255)**  **(770)** | **(20/24, 5-15.5/5-19)** | | **(55/265, 2.7-19.9/2.3-19.9)** |  |  |
| 12 | **(3691)**  **(1665)** | **(36/146, 2.5-14.8/2.2-16.3)** | | **(45/202, 2.7-14.1/1.5-14.5)** | **20, 4.5-18.5** |  |
| 13 | **(3561)**  **(2048)** | **(32/162, 1.4-9.9/1.1-9.9)** | | **(24/89,2.9-10.9/1.7-10.9)** | **21, 2.1-8.8** |  |
| 14 | **(3771)**  **(1822)** | **(138/681, 2-13.7/1.3-14.5)** | |  |  |  |
| 15 | **(2574)**  **(1110)** | **(21/61, 3-18.1/3-20)** | | **(42/176, 2.8-13.1/2.8-14.7)** | **14, 7.3-16.4** | **39, 1.2-2.1** |
| 16 | **(3729)**  **(1083)** | **(20/41, 7.3-10.9/5-10.9)** | | **(57/240, 3.3-10.5/2.5-10.5)** |  | **6; 2.2-3** |
| 17 | **(4372)**  **(864)** | **(28/140, 2.6-9.8/2.1-9.8)** | | **(20/46, 2.6-6.5/1.5-6.7)** |  | **96, 3.9-7.3** |
| 18 | **(2499)**  **(1316)** | **(54/266, 2.1-16.7/1.0-16.7)** | | **(21/74, 2.3-6.7/2.3-7)** | **36, 3.6-15** |  |
| 19 | **(3667)**  **(2017)** | | **(98/488, 2.3-13.7/2.3-14)** | **(44/172, 3.9-14.2/3.1-14.2)** | **15, 12-15.3** |  |
| 20 | **(2215)**  **(1095)** | | **(43/185, 4-16.7/4-16.7)** | **(55/227, 3.7-13.1/1.9-13.1)** |  |  |

**Recording time is the duration from discontinuing the neuromuscular blocking agent to the end of the recording, while count time is the interval from the onset of consistent active breathing until the recording's end. ∆P_es_: esophageal pressure swing. Case 1 and 3 with scarce reverse triggering breaths (7 and 4 only) and these reverse triggering breaths were not included for analysis.**
