# Supplementary material for: Pendelluft in patients with acute respiratory distress syndrome during trigger and reverse triggering breaths
Source: Sci Rep. 2023 Dec 13;13:22143. doi: 10.1038/s41598-023-49038-9 (PMC10719360; doi:10.1038/s41598-023-49038-9)
Supplement: Supplementary file 5 — Supplementary Table 2. [file 41598_2023_49038_MOESM5_ESM.docx]

| **Table 2E Performance of P_mus_ in the prediction of pendelluft volume in triggered breaths (n=1135)** | | | | | | | |
| --- | --- | --- | --- | --- | --- | --- | --- |
| **pendelluft (ml)** | **P_mus_ threshold (cmH_2_O)** | **sensitivity** | **specificity** | **PPV** | **NPV** | **AUC** |  |
| **5.0** | **8.68** | **0.78** | **0.40** | **0.29** | **0.85** | **0.584** | **P < 0.0001** |
| **10.0** | **10.10** | **0.71** | **0.50** | **0.13** | **0.94** | **0.587** | **P = 0.002** |
| **15.0** | **10.61** | **0.72** | **0.54** | **0.09** | **0.97** | **0.623** | **P = 0.001** |
| **20.0** | **10.61** | **0.76** | **0.54** | **0.07** | **0.98** | **0.641** | **P < 0.001** |
| **25.0** | **10.61** | **0.78** | **0.54** | **0.05** | **0.99** | **0.653** | **P = 0.002** |

**P_mus_: inspiratory muscular pressure**；**PPV: positive predictive value； NPV: negative predictive value.**

**AUC: area under curve.**
